# Aripiprazole once-monthly for the treatment of adult patients with earlier-stage bipolar I disorder: a *post hoc* analysis of data from a double-blind, placebo-controlled, 52-week randomized withdrawal trial

**DOI:** 10.1186/s40345-024-00358-3

**Published:** 2024-10-27

**Authors:** Karimah S. Bell Lynum, Christine F. Castro, Zhen Zhang, Mehul Patel, Mauricio Tohen

**Affiliations:** 1 Otsuka Pharmaceutical Development & Commercialization, Inc., Princeton, NJ USA; 2 Lundbeck LLC, Deerfield, IL USA; 3 Department of Psychiatry & Behavioral Sciences, University of New Mexico, Albuquerque, NM USA

**Keywords:** Aripiprazole, Bipolar disorder, Safety, Treatment outcome

## Abstract

**Background:**

Increased awareness of the factors contributing to the diagnostic disparities seen in bipolar disorder between individuals of different heritage is needed to achieve equity in diagnosis and treatment. One such inequity is the provision of earlier treatment. Earlier treatment of patients diagnosed with bipolar disorder may prolong time to recurrence of mood episodes and reduce functional impairment and other poor outcomes associated with disease progression. The aim of this *post hoc* analysis was to study the efficacy and safety of long-acting injectable aripiprazole once-monthly 400 mg (AOM 400) in patients with earlier-stage bipolar I disorder (BP-I). Data from a 52-week multicenter, double-blind, placebo-controlled, randomized withdrawal trial of AOM 400 versus placebo in patients with BP‑I (NCT01567527) were analyzed. Those patients in the lowest quartiles for age (18–≤32 years; n = 70) or disease duration (0.13–≤4.6 years; n = 67) at baseline were categorized with earlier-stage BP-I. The primary endpoint was time from randomization to recurrence of any mood episode. Other endpoints included proportion of patients with recurrence of any mood episode, and change from baseline in Young Mania Rating Scale (YMRS) and Montgomery–Åsberg Depression Rating Scale (MADRS) total scores.

**Results:**

Maintenance treatment with AOM 400 significantly delayed time to recurrence of any mood episode versus placebo in patients aged 18–≤32 years (hazard ratio [HR]: 2.46 [95% confidence interval (CI) 1.09, 5.55]; p = 0.0251) or with disease duration 0.13–≤4.6 years (HR: 3.21 [95% CI 1.35, 7.65]; p = 0.005). This was largely driven by a lower proportion of patients in the AOM 400 group with YMRS total score ≥15 or clinical worsening. Changes from baseline in MADRS total score in both earlier-stage groups indicated AOM 400 did not worsen depression versus placebo. The safety profile of AOM 400 was consistent with the original study. Note that the original study included patients who had previously been stabilized on AOM 400 monotherapy, which may have enriched the population with patients who respond to and tolerate AOM 400.

**Conclusions:**

In this *post hoc* analysis, AOM 400 prolonged time to recurrence of any mood episode versus placebo in earlier-stage BP-I. These findings support early initiation of maintenance treatment with AOM 400.

**Supplementary Information:**

The online version contains supplementary material available at 10.1186/s40345-024-00358-3.

## Background

Bipolar I disorder (BP‑I) is a lifelong illness, characterized by episodes of mania, hypomania, and depression (American Psychiatric Association 2022; Tohen et al. 2020). Globally, the pooled lifetime prevalence of BP-I is 1.06% (Clemente et al. 2015); in the United States (US), the lifetime prevalence is 2.1% (Blanco et al. 2017). Recurrence of mood episodes in patients diagnosed with BP-I is associated with poor outcomes, including functional impairment, psychiatric and medical co-morbidity, and an increased risk of suicidality, disability, unemployment, and hospitalization (Peters et al. 2016). Despite the burden of the disease, there are currently no diagnostic tests or biomarkers for bipolar disorder (Craddock and Sklar 2013; Vöhringer and Perlis 2016) – definition of its phenotype is based purely on clinical features (American Psychiatric Association 2022). Diagnosis is further complicated by the clinical features of bipolar disorder (both BP-I and bipolar II disorder [BP-II]) overlapping with those of other psychiatric conditions (Brenner and Shyn 2014), particularly major depressive disorder (Vöhringer and Perlis 2016).

Clinical guidelines recommend the use of antipsychotics with or without mood stabilizers for the maintenance treatment of BP-I (American Psychiatric Association 2010; Verdolini et al. 2021; Yatham et al. 2018) to reduce the likelihood of symptom recurrence and relapse (American Psychiatric Association 2010; Yatham et al. 2018). It should be noted that first-generation antipsychotics have been associated with extrapyramidal symptoms and reports of neurotoxicity, and may worsen depression (Narasimhan et al. 2007); second-generation antipsychotics, such as risperidone and aripiprazole, have been associated with neuroprotective effects (Chen and Nasrallah 2019; Nasrallah and Chen 2017). Compared with oral antipsychotics, long-acting injectable (LAI) formulations have been associated with improved treatment adherence (Yan et al. 2018) and beneficial clinical outcomes such as fewer emergency department visits and shorter hospital stays (Bartoli et al. 2022). In the US, LAI formulations of the second-generation antipsychotics risperidone (available in dosage strengths of 12.5 mg, 25 mg, 37.5 mg, and 50 mg, to be administered once every two weeks) (Risperdal Consta^®^ [risperidone] 2021) and aripiprazole (available as either aripiprazole once-monthly 400 mg [AOM 400] or aripiprazole 2-month ready-to-use 960 mg [Ari 2MRTU 960]) (Abilify Asimtufii^®^ [aripiprazole] 2023; Abilify Maintena^®^ [aripiprazole] 2020), are approved for the maintenance monotherapy treatment of BP‑I in adults (Risperdal Consta^® ^[risperidone] 2021; Abilify Asimtufii^®^ [aripiprazole] 2023; Abilify Maintena^®^ [aripiprazole] 2020). Risperidone is also approved as adjunctive therapy to lithium or valproate for the maintenance treatment of BP-I in adults in the US (Risperdal Consta^®^ [risperidone] 2021).

The approval of AOM 400 for the maintenance treatment of BP‑I was based on data from an international, double-blind, placebo-controlled, 52-week randomized withdrawal trial (NCT01567527; https://www.clinicaltrials.gov; trial registration date: 26 March 2012) (Abilify Maintena^®^ [aripiprazole] 2020; Calabrese et al. 2017). In this trial, AOM 400 significantly delayed the time to recurrence of any mood episode compared with placebo (Calabrese et al. 2017). However, despite the established efficacy of LAI antipsychotics, including AOM 400, for the maintenance treatment of BP‑I (Calabrese et al. 2017; Quiroz et al. 2010; Vieta et al. 2012), usual practice is to reserve LAIs for non-adherent patients who have experienced multiple mood episodes (Tohen et al. 2020). An analysis of medication use patterns between 1996 and 2018 found that LAIs were markedly underutilized as compared to their counterpart oral formulations over a 5-year follow-up period in patients with newly diagnosed bipolar disorder (Poranen et al. 2022). This is despite the hypothesis that LAI antipsychotics could, potentially, play a role in protecting against progressive functional deterioration if used earlier in the course of illness (Tohen et al. 2020; Stip et al. 2020). Only one study has been identified that investigated the efficacy and safety of LAIs early in the course of bipolar disorder (Lähteenvuo et al. 2023). This study found that LAI antipsychotics were associated with a decreased risk of hospital admission for psychiatric reasons in patients with first-episode bipolar disorder (Lähteenvuo et al. 2023).

Classification of ‘early’ bipolar disorder varies in the literature (Chakrabarty et al. 2019; Cirone et al. 2021; Cotton et al. 2019; Patel et al. 2021; Ratheesh et al. 2023; Wong et al. 2021), with definitions based on time since the first manic episode (Chakrabarty et al. 2019), age at onset (Cirone et al. 2021), number of untreated/treated manic episodes (Ratheesh et al. 2023), length of treatment (Wong et al. 2021), and number of lifetime bipolar medications (Patel et al. 2021). This *post hoc* analysis defined earlier-stage BP-I according to patient age or disease duration, and aimed to evaluate the efficacy and safety of AOM 400 in patients with earlier-stage BP-I using data from the randomized withdrawal trial of AOM 400 versus placebo (NCT01567527).

## Materials and methods

### Study design and patients

This was a *post hoc* analysis performed on data from a 52-week multicenter, double-blind, placebo-controlled, randomized withdrawal trial of AOM 400 versus placebo in patients diagnosed with BP‑I (NCT01567527; https://www.clinicaltrials.gov; trial registration date: 26 March 2012) (Calabrese et al. 2017). The trial was conducted in accordance with the International Conference of Harmonization and Good Clinical Practice consolidated guideline and approved by the relevant institutional review board (IRB), with informed consent obtained for all patients. All IRBs are listed in Supplementary Table S1. The results of the trial have been published previously (Calabrese et al. 2017). In brief, the trial consisted of four treatment phases: a phase to transition patients onto oral aripiprazole (4–6 weeks), an oral aripiprazole stabilization phase (2–8 weeks), an AOM 400 stabilization phase (12–28 weeks), and a randomized, double-blind, placebo-controlled phase (52 weeks). Patients were eligible to be included if they were aged 18–65 years, had been diagnosed with BP-I according to the Diagnostic and Statistical Manual of Mental Disorders, Fourth Edition, Text Revision (DSM-IV-TR), had experienced one or more previous manic or mixed episodes with manic symptoms of sufficient severity to require hospitalization or treatment with a mood stabilizer/antipsychotic agent, and were currently experiencing a manic episode (according to DSM-IV-TR criteria) with a Young Mania Rating Scale (YMRS) total score ≥20. It should be noted that, though the diagnostic criteria for BP-I and manic episodes were based on DSM-IV-TR criteria in the study (American Psychiatric Association 2000), the criteria have since been superseded by the Diagnostic and Statistical Manual of Mental Disorders, Fifth Edition, Text Revision (DSM-V-TR) (American Psychiatric Association 2022). Patients were excluded if they had experienced ≥9 episodes in the past year (i.e., rapid-cycling BP-I).

In this *post hoc* analysis, patients from the 52-week trial were categorized as having earlier-stage BP-I if they were included in the first (i.e., lowest) quartile for age or disease duration at study entry, based on data from the overall patient population. The use of quartiles to define subgroups for analyses is common in the literature (Grewal et al. 2022; He et al. 2022, 2023; Innala et al. 2014; Shi et al. 2022).

### Outcomes

Consistent with the original study, the primary efficacy endpoint was time from randomization to recurrence of any mood episode, defined as meeting any of the following predetermined criteria: YMRS total score ≥15, Montgomery–Åsberg Depression Rating Scale (MADRS) total score ≥15, Clinical Global Impression for Bipolar Disorder – Severity (CGI-BP-S) score >4, a serious adverse event (AE) of worsening BP-I, hospitalization for any mood episode, discontinuation due to lack of efficacy/AE of worsening BP-I, clinical worsening requiring additional treatment (use of a mood stabilizer, antidepressant treatment, antipsychotic medication, or an increase in benzodiazepine dose above the highest permitted dose), or active suicidality (defined as a score of ≥4 on MADRS item 10 or “yes” in answer to question 4 or 5 on the Columbia Suicide Severity Rating Scale). The key secondary endpoint was the proportion of patients meeting any of the aforementioned criteria for recurrence of any mood episode. Change from randomization in YMRS and MADRS total scores were included as additional endpoints. Safety assessments were also conducted through treatment-emergent AE (TEAE) reporting.

### Statistical methods

All analyses compared AOM 400 with placebo. The safety sample included all patients who received at least one injection in the randomized, double-blind, placebo-controlled phase of NCT01567527, and the efficacy sample included those who received at least one injection and had at least one post-baseline efficacy assessment in this phase. Baseline characteristics and safety data were summarized descriptively. Time to recurrence for each of the earlier BP‑I subgroups was plotted as a Kaplan–Meier curve and analyzed using the log-rank test; hazard ratios (HR; AOM 400 versus placebo) and 95% confidence intervals (CIs) were estimated using a Cox proportional hazards model, with treatment as term. The proportion of patients meeting each of the recurrence criteria was analyzed using Fisher’s exact test. Changes from baseline in YMRS and MADRS total scores were analyzed using mixed model for repeated measures (MMRM), with treatment, region, trial week, and treatment-by-week interaction as terms, as well as the covariates of baseline‑score‑by‑week interaction. For all tests, p-values <0.05 were deemed statistically significant. Analyses were performed using SAS software (version 9.4) (SAS Institute Inc., Cary, NC, USA).

## Results

### Patient disposition and baseline characteristics

**Fig. 1:**
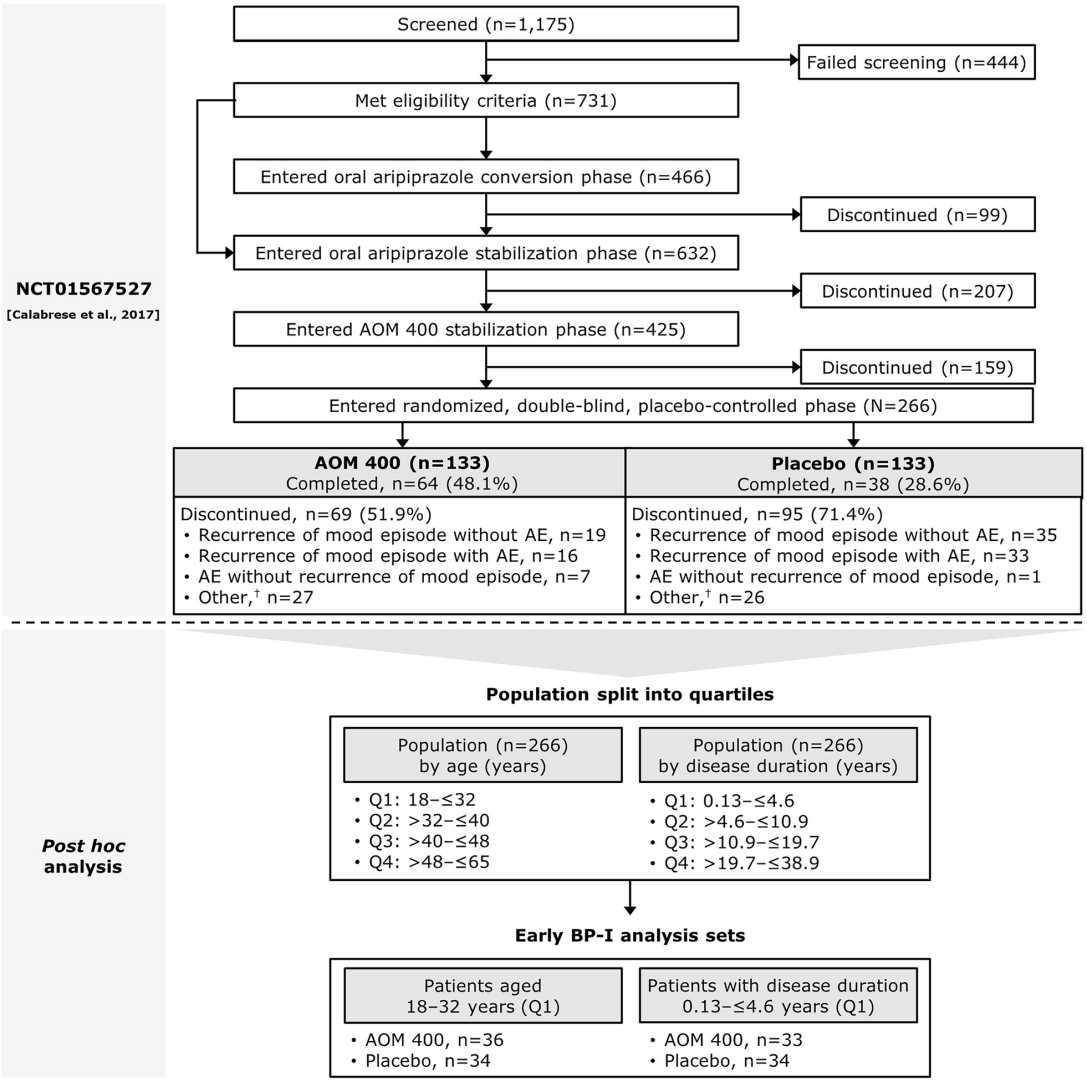
Patient disposition. ^†^Discontinuation due to one of the following: patient withdrew consent, patient lost to follow-up, patient met withdrawal criteria, sponsor discontinued patient from study, or protocol deviation. AE, adverse event; AOM 400, aripiprazole once-monthly 400 mg; BP-I, bipolar I disorder; Q, quartile. This figure is adapted with permission from Figure 1 of Calabrese et al. J Clin Psychiatry 2017. Changes include editing, formatting, and the addition of original content. The source publication is available at https://doi.org/10.4088/JCP.16m11201

Patient disposition is shown in Fig. 1. When the overall population that entered the randomized, double-blind, placebo-controlled phase of the 52-week trial was split into quartiles by age, patients in the first quartile were aged 18–32 years. When the same overall patient population of the 52-week trial was split into quartiles by disease duration, patients in the first quartile had a disease duration of 0.13–≤4.6 years.

Baseline demographics and clinical characteristics of patients with earlier-stage BP-I, defined according to patient age or disease duration at baseline, are shown in Table 1.

**Table 1:** Baseline demographics and clinical characteristics of patients from NCT01567527 with earlier stages of BP-I

Baseline demographics	Earlier BP-I population according to age (18–≤32 years old)		Earlier BP-I population according to disease duration (0.13–≤4.6 years)	
	AOM 400 (n=36)	Placebo (n=34)	AOM 400 (n=33)	Placebo (n=34)
Age (years), mean (SD) [range]	27.7 (3.1) [21–33]	25.9 (4.1) [19–33]	36.9 (10.5) [22–63]	33.9 (11.5) [19–59]
Female, n (%)	29 (80.6)	15 (44.1)	22 (66.7)	17 (50.0)
Race, n (%)				
Asian	5 (13.9)	3 (8.8)	10 (30.3)	10 (29.4)
Black or African American	10 (27.8)	7 (20.6)	8 (24.2)	8 (23.5)
Other	1 (2.8)	3 (8.8)	2 (6.1)	2 (5.9)
White	20 (55.6)	21 (61.8)	13 (39.4)	14 (41.2)
Ethnicity				
Hispanic or Latino	4 (11.1)	3 (8.8)	3 (9.1)	1 (2.9)
Non-Hispanic or Latino	32 (88.9)	31 (91.2)	30 (90.9)	33 (97.1)
BMI (kg/m^2^), mean (SD) [range]	31.6 (8.8) [17.5–54.0]	31.9 (7.7) [18.7–55.1]	30.9 (7.0) [19.9–51.8]	28.6 (5.1) [18.7–41.5]
Clinical characteristics				
Age (years), mean (SD) [range] at:				
First manic episode	19.8 (4.2) [13–30]	18.1 (4.1) [9–28]	28.4 (10.5) [12–60]	29.5 (12.6) [13–59]
First mixed episode	20.4 (4.8) [14–30]	17.4 (4.7) [10–25]	28.4 (11.0) [15–53]	27.9 (10.0) [14–48]
First depressive episode	19.5 (4.8) [12–30]	17.4 (4.0) [10–24]	25.8 (8.1) [13–44]	23.4 (9.2) [7–45]
First BP-I diagnosis	20.2 (4.3) [13–30]	18.5 (4.7) [9–28]	34.1 (10.5) [20–60]	31.1 (11.4) [16–54]
Duration of disease prior to enrollment (years), mean (SD) [range]	7.1 (4.8) [0.5–17.7]	6.9 (5.5) [0.4–18]	2.5 (1.2) [0.5–4.5]	2.3 (1.3) [0.1–4.6]
Duration of current manic episode (years), mean (SD) [range]	0.2 (0.2) [0.1–1.2]	0.2 (0.1) [0–0.7]	0.2 (0.2) [0.1–1.2]	0.2 (0.2) [0–0.7]
Number of mood episodes, mean (SD) [range]				
Past 12 months	2.4 (1.4) [1–8]	2.4 (1.1) [1–5]	2.6 (1.2) [1–7]	2.2 (1.0) [1–5]
Last 10 years	11.3 (10.2) [2–50]	9.6 (8.6) [3–40]	11.1 (12.6) [2–70]	5.5 (4.4) [2–25]
Number of lifetime episodes, mean (SD) [range]				
Manic	10.5 (18.6) [1–100]	5.9 (5.5) [2–25]	12.1 (41.2) [1–240]	3.9 (3.1) [2–15]
Mixed	3.4 (6.7) [0–30]	2.1 (3.8) [0–15]	1.6 (4.5) [0–25]	1.0 (2.4) [0–12]
Depressive	7.8 (16.9) [0–100]	4.1 (5.1) [0–25]	5.4 (6.7) [0–27]	2.6 (3.1) [0–15]
Number of prior hospitalizations for a mood episode, mean (SD) [range]	2.7 (3.0) [0–12]	2.1 (2.2) [0–10]	1.5 (1.8) [0–8]	2.0 (2.5) [0–14]
YMRS total score, mean (SD) [range]	3.5 (3.7) [0–12]	2.7 (3.3) [0–12]	1.8 (3.0) [0–12]	2.3 (2.7) [0–12]
MADRS total score, mean (SD) [range]	2.6 (3.1) [0–11]	2.6 (3.1) [0–10]	2.2 (2.6) [0–8]	2.5 (3.4) [0–11]
CGI-BP-S score, mean (SD) [range]				
Mania	1.6 (0.8) [1–4]	1.4 (0.6) [1–3]	1.3 (0.5) [1–3]	1.3 (0.5) [1–3]
Depression	1.4 (0.6) [1–3]	1.3 (0.6) [1–3]	1.4 (0.5) [1–2]	1.3 (0.5) [1–3]
Overall	1.8 (0.7) [1–4]	1.6 (0.7) [1–3]	1.5 (0.6) [1–3]	1.4 (0.6) [1–3]

Baseline was defined as the last visit in the AOM 400 stabilization phase of the trial

AOM 400, aripiprazole once-monthly 400 mg; BMI, body mass index; BP-I, bipolar I disorder; CGI-BP-S, Clinical Global Impression for Bipolar Disorder – Severity; MADRS, Montgomery–Åsberg Depression Rating Scale; SD, standard deviation; YMRS, Young Mania Rating Scale

### Efficacy outcomes in patients with earlier-stage BP-I, defined by patient age at baseline

**Fig. 2:**
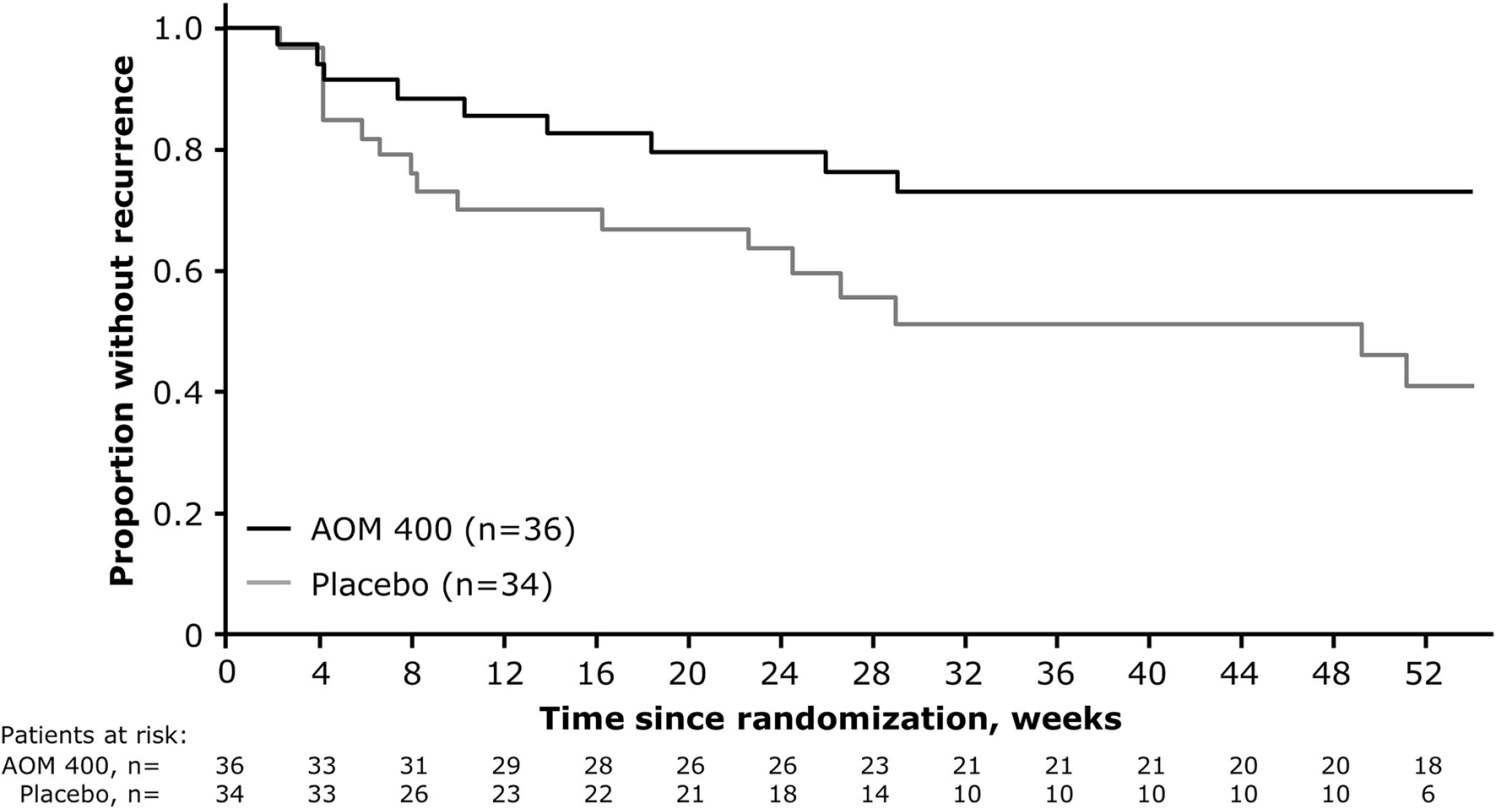
Time from randomization to recurrence of any mood episode in patients aged 18–32 years. AOM 400, aripiprazole once-monthly 400 mg

For patients aged 18–32 years, time to recurrence of any mood episode was significantly delayed with AOM 400 versus placebo, with the risk of recurrence 2.46-times lower with AOM 400 compared with placebo (HR: 2.46 [95% CI 1.09, 5.55]; p = 0.0251) (Fig. 2).

The benefit of AOM 400 over placebo was driven by a significantly lower proportion of patients aged 18–32 years experiencing a YMRS total score ≥15 (5.6% [2/36] versus 44.1% [15/34], respectively; p = 0.0002) or clinical worsening requiring additional treatment (8.3% [3/36] versus 38.2% [13/34], respectively; p = 0.004) (Table 2).

**Table 2:** Proportion of patients aged 18–32 years meeting each of the criteria for recurrence of any mood episode

Criteria for recurrence of mood episode	AOM 400 (n=36)	Placebo (n=34)	P-value^a^
Any, n (%)	9 (25)	17 (50)	0.0472
Hospitalization for any mood episode, n (%)	1 (2.8)	4 (11.8)	0.1921
YMRS total score ≥15, n (%)	2 (5.6)	15 (44.1)	0.0002
MADRS total score ≥15, n (%)	6 (16.7)	4 (11.8)	0.7356
CGI-BP-S score >4, n (%)	1 (2.8)	4 (11.8)	0.1921
SAE of worsening BP-I, n (%)	1 (2.8)	4 (11.8)	0.1921
Discontinuation due to lack of efficacy or AE of worsening BP-I, n (%)	6 (16.7)	9 (26.5)	0.3888
Clinical worsening requiring additional treatment,^b^ n (%)	3 (8.3)	13 (38.2)	0.004
Active suicidality, n (%)	1 (2.8)	1 (2.9)	1

AE, adverse event; AOM 400, aripiprazole once-monthly 400 mg; BP-I, bipolar I disorder; CGI-BP-S, Clinical Global Impression for Bipolar Disorder – Severity; MADRS, Montgomery–Åsberg Depression Rating Scale; SAE, serious adverse event; YMRS, Young Mania Rating Scale

^a^Calculated using Fisher’s exact test

^b^Additional treatment comprised mood stabilizers, antidepressant treatment, antipsychotic medication, or an increase in benzodiazepine dose above the highest permitted dose

**Fig. 3:**
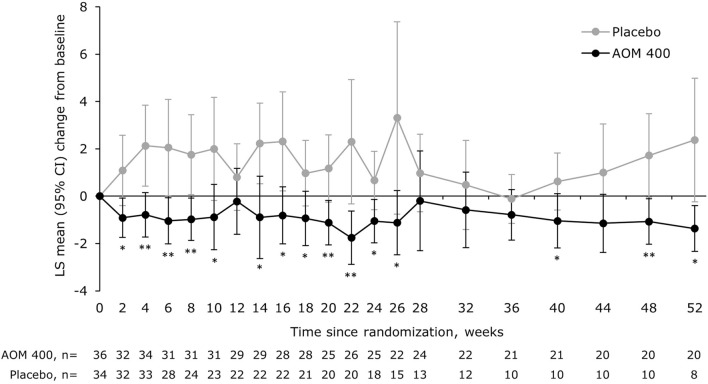
LS mean change from baseline in YMRS total score in patients aged 18–32 years. *p<0.05, **p<0.01 versus placebo (MMRM with treatment, region, trial week, and treatment-by-week interaction as terms, as well as the covariates of baseline‑score‑by‑week interaction). AOM 400, aripiprazole once-monthly 400 mg; CI, confidence interval; LS, least squares; MMRM, mixed model for repeated measures; YMRS, Young Mania Rating Scale

The mean change from baseline in YMRS total score significantly favored AOM 400 over placebo at multiple time points, with the efficacy of AOM 400 sustained over time (Fig. 3). Notably, from Week 36 to 52, YMRS total score remained stable with AOM 400, indicating ongoing improvement in manic symptoms; in contrast, in the placebo group, YMRS total score increased over the same time period. The observed separation between AOM 400 and placebo was consistent with the lower event rate for a YMRS total score ≥15 with AOM 400 (5.6%) versus placebo (44.1%).

**Fig. 4:**
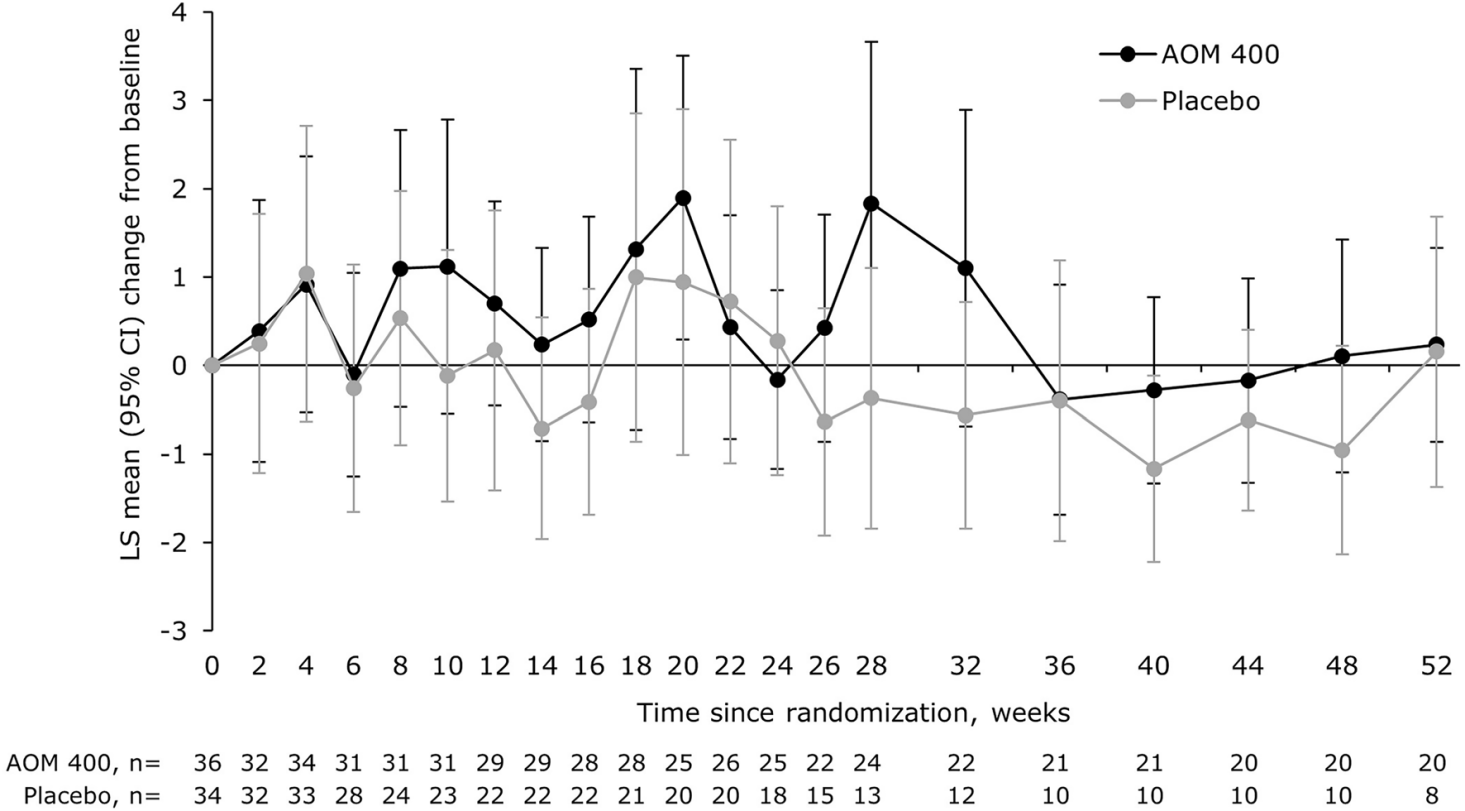
LS mean change from baseline in MADRS total score in patients aged 18–32 years. MMRM with treatment, region, trial week, and treatment-by-week interaction as terms, as well as the covariates of baseline‑score‑by‑week interaction. AOM 400, aripiprazole once-monthly 400 mg; CI, confidence interval; LS, least squares; MADRS, Montgomery–Åsberg Depression Rating Scale; MMRM, mixed model for repeated measures

There was no significant between-treatment difference in MADRS total score (Fig. 4).

### Efficacy outcomes in patients with earlier-stage BP-I, defined by disease duration at baseline

**Fig. 5:**
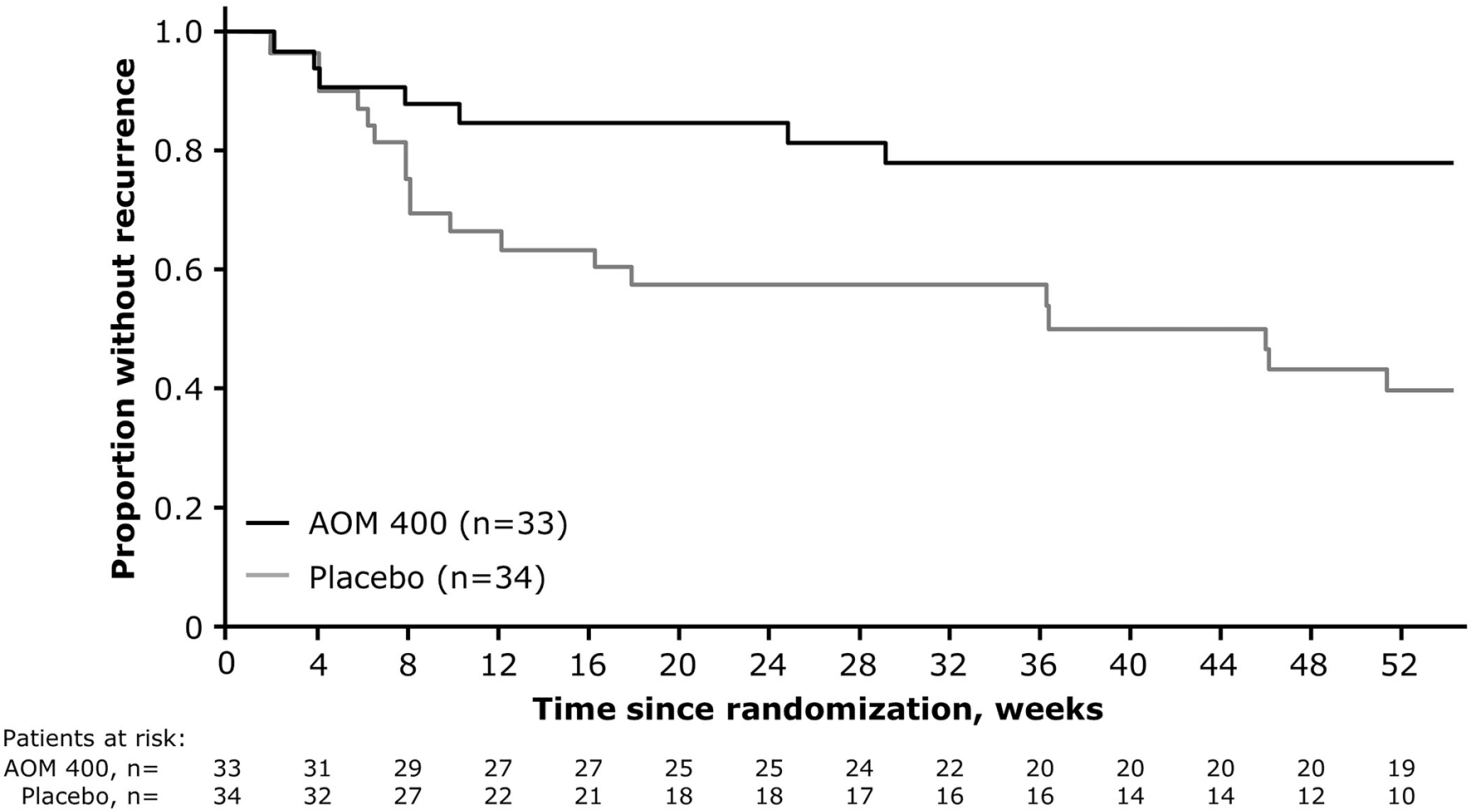
Time from randomization to recurrence of any mood episode in patients with a disease duration of 0.13–≤4.6 years. AOM 400, aripiprazole once-monthly 400 mg

For patients with a disease duration of 0.13–≤4.6 years, time to recurrence of any mood episode was significantly delayed with AOM 400 versus placebo, with the risk of recurrence 3.21-times lower with AOM 400 compared with placebo (HR: 3.21 [95% CI 1.35, 7.65]; p = 0.005) (Fig. 5).

The benefit of AOM 400 over placebo was driven by a significantly lower proportion of patients with a disease duration 0.13–≤4.6 years experiencing a YMRS total score ≥15 (6.1% [2/33] versus 41.2% [14/34], respectively; p = 0.0012) or clinical worsening requiring additional treatment (6.1% [2/33] versus 38.2% [13/34], respectively; p = 0.0026) (Table 3).

**Table 3:** Proportion of patients with a disease duration of 0.13–≤4.6 years meeting each of the criteria for recurrence of any mood episode

Criteria for recurrence of mood episode	AOM 400 (n=33)	Placebo (n=34)	P-value^a^
Any, n (%)	7 (21.2)	19 (55.9)	0.0055
Hospitalization for any mood episode, n (%)	1 (3.0)	4 (11.8)	0.3559
YMRS total score ≥15, n (%)	2 (6.1)	14 (41.2)	0.0012
MADRS total score ≥15, n (%)	5 (15.2)	4 (11.8)	0.7337
CGI-BP-S score >4, n (%)	2 (6.1)	4 (11.8)	0.6728
SAE of worsening BP-I, n (%)	1 (3.0)	4 (11.8)	0.3559
Discontinuation due to lack of efficacy or AE of worsening BP-I, n (%)	7 (21.2)	11 (32.4)	0.4101
Clinical worsening requiring additional treatment,^b^ n (%)	2 (6.1)	13 (38.2)	0.0026
Active suicidality, n (%)	0 (0.0)	0 (0.0)	–

AE, adverse event; AOM 400, aripiprazole once-monthly 400 mg; BP-I, bipolar I disorder; CGI-BP-S, Clinical Global Impression for Bipolar Disorder – Severity; MADRS, Montgomery–Åsberg Depression Rating Scale; SAE, serious adverse event; YMRS, Young Mania Rating Scale

^a^Calculated using Fisher’s exact test

^b^Additional treatment comprised mood stabilizers, antidepressant treatment, antipsychotic medication, or an increase in benzodiazepine dose above the highest permitted dose

**Fig. 6:**
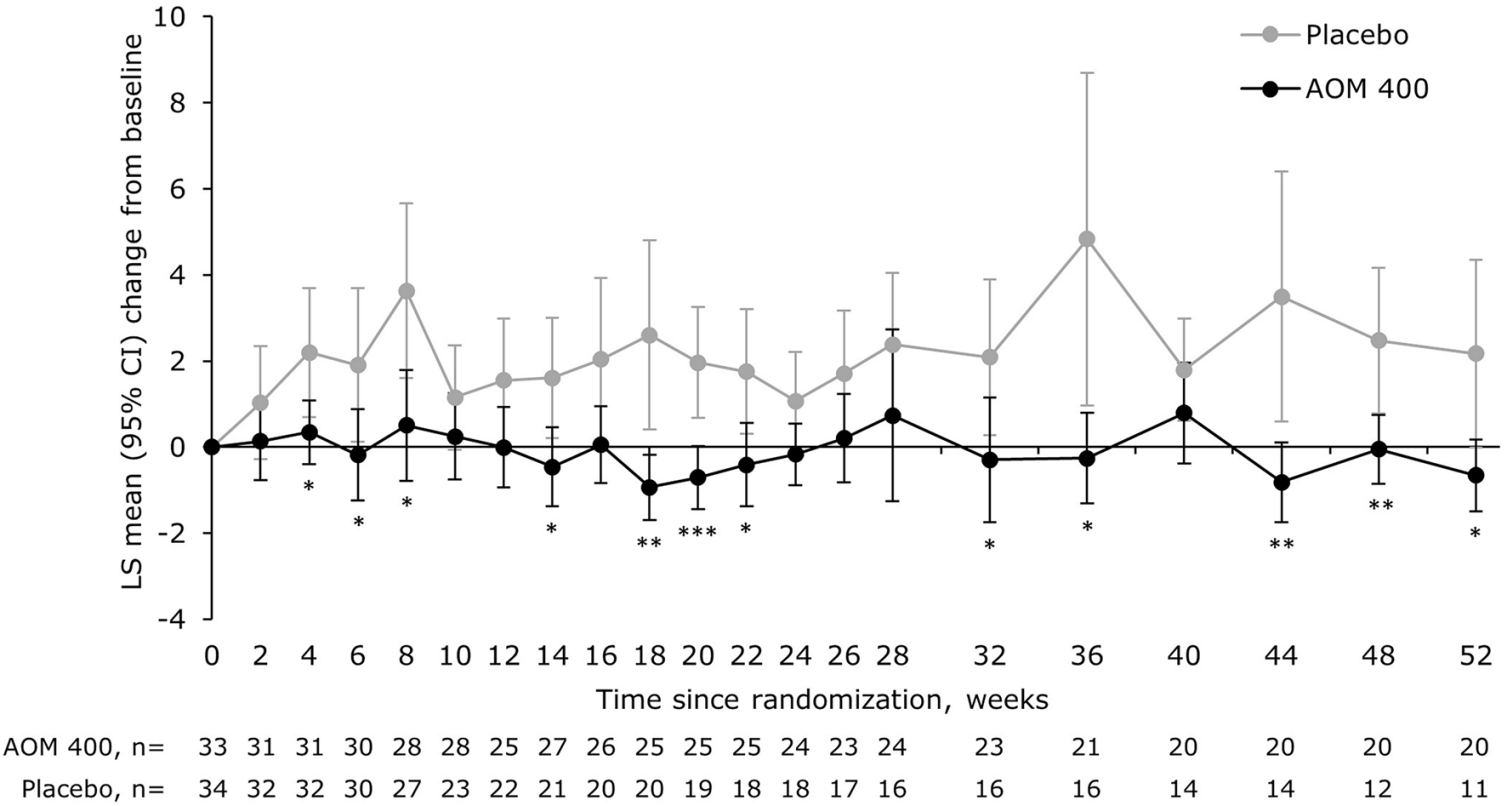
LS mean change from baseline in YMRS total score in patients with a disease duration of 0.13–≤4.6 years. *p<0.05, **p<0.01, ***p<0.001 versus placebo (MMRM with treatment, region, trial week, and treatment-by-week interaction as terms, as well as the covariates of baseline‑score‑by‑week interaction). AOM 400, aripiprazole once-monthly 400 mg; CI, confidence interval; LS, least squares; MMRM, mixed model for repeated measures; YMRS, Young Mania Rating Scale

The mean change from baseline in YMRS total score significantly favored AOM 400 over placebo at multiple time points, with the efficacy of AOM 400 sustained over time (Fig. 6). The observed separation between AOM 400 and placebo was consistent with the lower event rate for a YMRS total score of ≥15 with AOM 400 (6.1%) versus placebo (41.2%).

**Fig. 7:**
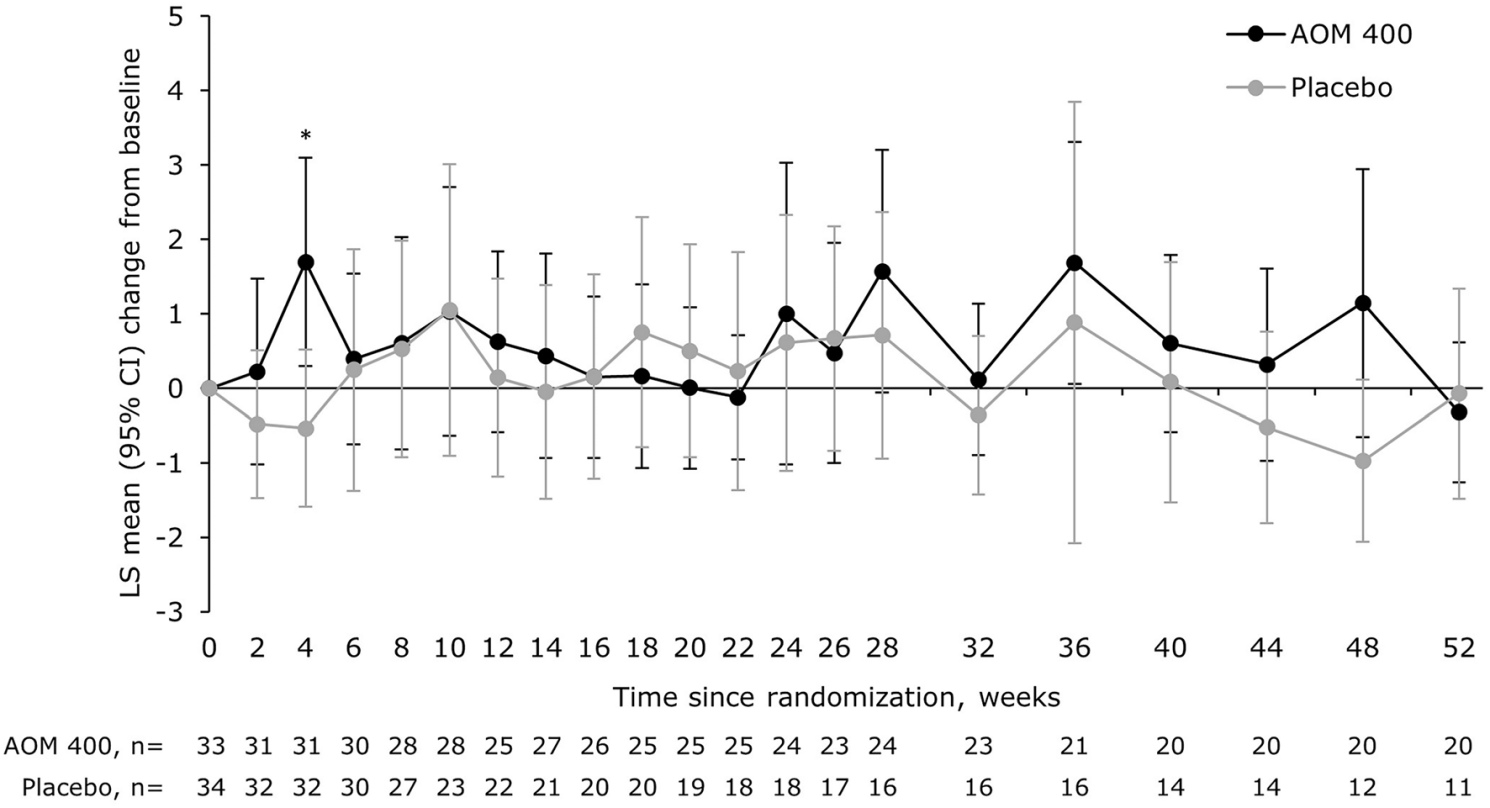
LS mean change from baseline in MADRS total score in patients with a disease duration of 0.13–≤4.6 years. *p<0.05 versus placebo (MMRM with treatment, region, trial week, and treatment-by-week interaction as terms, as well as the covariates of baseline‑score‑by‑week interaction). AOM 400, aripiprazole once-monthly 400 mg; CI, confidence interval; LS, least squares; MADRS, Montgomery–Åsberg Depression Rating Scale; MMRM, mixed model for repeated measures

There was no significant between-treatment difference in MADRS total score at Week 52 (Fig. 7).

### Safety

TEAEs experienced by patients with earlier-stage BP-I are shown in Table 4. Increased weight and akathisia were the two most common TEAEs (incidence of ≥5%) across both treatment groups, regardless of whether the earlier-stage BP-I population was defined according to age or disease duration at baseline.

**Table 4:** TEAEs in patients from NCT01567527 with earlier-stage BP-I

TEAE, n (%)	Earlier BP-I population according to age (18–≤32 years old)		Earlier BP-I population according to disease duration (0.13–≤4.6 years)	
	AOM 400 (n=36)	Placebo (n=34)	AOM 400 (n=33)	Placebo (n=34)
Any	26 (72.2)	28 (82.4)	25 (75.8)	27 (79.4)
Weight increased	10 (27.8)	11 (32.4)	8 (24.2)	7 (20.6)
Akathisia	7 (19.4)	6 (17.6)	7 (21.2)	5 (14.7)
Tremor	0 (0.0)	1 (2.9)	1 (3.0)	1 (2.9)
Insomnia	4 (11.1)	2 (5.9)	3 (9.1)	2 (5.9)
Depression	2 (5.6)	0 (0.0)	3 (9.1)	0 (0.0)
Sinusitis	3 (8.3)	0 (0.0)	2 (6.1)	0 (0.0)
Vision blurred	3 (8.3)	0 (0.0)	2 (6.1)	0 (0.0)
Dry mouth	0 (0.0)	0 (0.0)	2 (6.1)	0 (0.0)
Libido decreased	1 (2.8)	2 (5.9)	0 (0.0)	2 (5.9)
Anemia	2 (5.6)	0 (0.0)	1 (3.0)	0 (0.0)
Anxiety	2 (5.6)	0 (0.0)	1 (3.0)	1 (2.9)
Bronchitis	2 (5.6)	0 (0.0)	0 (0.0)	0 (0.0)
Somnolence	2 (5.6)	1 (2.9)	1 (3.0)	0 (0.0)
Blood creatine phosphokinase increased	0 (0.0)	0 (0.0)	1 (3.0)	0 (0.0)
Constipation	0 (0.0)	0 (0.0)	1 (3.0)	1 (2.9)
Increased appetite	0 (0.0)	0 (0.0)	1 (3.0)	0 (0.0)
Procedural pain	1 (2.8)	0 (0.0)	1 (3.0)	1 (2.9)
Restlessness	1 (2.8)	0 (0.0)	1 (3.0)	0 (0.0)
Salivary hypersecretion	0 (0.0)	0 (0.0)	1 (3.0)	0 (0.0)
Bipolar disorder	1 (2.8)	0 (0.0)	0 (0.0)	1 (2.9)
Urinary tract infection	1 (2.8)	1 (2.9)	0 (0.0)	0 (0.0)
Influenza	0 (0.0)	0 (0.0)	0 (0.0)	0 (0.0)
TEAEs related to EPS				
Akathisia events^a^	8 (22.2)	6 (17.6)	8 (24.2)	5 (14.7)
Parkinsonism events	1 (2.8)	2 (5.9)	2 (6.1)	2 (5.9)
Dyskinetic events	0 (0.0)	1 (2.9)	1 (3.0)	2 (5.9)
Dystonic events	1 (2.8)	0 (0.0)	1 (3.0)	0 (0.0)

AOM 400, aripiprazole once-monthly 400 mg; BP-I, bipolar I disorder; EPS, extrapyramidal symptoms; TEAE, treatment-emergent adverse event

^a^Includes akathisia, hyperkinesia, hyperkinesia neonatal, and psychomotor hyperactivity

## Discussion

There is no universal consensus on the definition of ‘early’ bipolar disorder, which has been variously defined in the literature according to medication use (Patel et al. 2021), age (Cirone et al. 2021), duration of illness (Chakrabarty et al. 2019; Wong et al. 2021), and number of lifetime mood episodes (Cotton et al. 2019; Ratheesh et al. 2023). As an example, in a systematic review of interventions early in the course of BP-I or BP-II, early disease was defined as seeking treatment for the first time for a manic episode, having a lifetime history of up to three manic episodes, or having a lifetime history of up to six mood episodes (Ratheesh et al. 2023). Alternatively, in a randomized controlled trial evaluating tailored psychological interventions in young patients (aged 15–25 years) diagnosed with BP-I, early disease was defined as having had at least one manic episode in the previous 2 years, with a lifetime history of no more than five treated/untreated manic or hypomanic episodes (Cotton et al. 2019). Despite the variety in definitions of early bipolar disorder, current evidence suggests that bipolar disorder is a progressive illness and should, therefore, be the target of early intervention strategies (Vieta et al. 2018). In this analysis, a statistical approach was used to define patients with earlier-stage BP-I, segmented into quartiles by age or disease duration at baseline, which is a method often used in the literature to categorize subpopulations across different therapy areas (Grewal et al. 2020; He et al. 2022, 2023; Innala et al. 2014; Shi et al. 2022).

Previously, a register-based national cohort study in 60,045 patients diagnosed with bipolar disorder evaluated the real-world effectiveness of antipsychotics and mood stabilizers by assessing the risk of psychiatric hospital admissions as a primary outcome (Lähteenvuo et al. 2023). The outcome was also assessed in a sub-cohort of 26,395 patients with first-episode bipolar disorder (Lähteenvuo et al. 2023). The study found that LAI antipsychotics were associated with a decreased risk of admission for psychiatric reasons – a finding that was evident both in the main cohort and in the sub-cohort of patients diagnosed with first-episode bipolar disorder (Lähteenvuo et al. 2023). This *post hoc* analysis using data from NCT01567527, however, is the first to have focused on investigating the use of AOM 400 in patients with earlier-stage BP-I specifically.

The data from this analysis show that use of AOM 400 as a maintenance treatment in patients with earlier-stage BP-I significantly prolonged the time to recurrence of any mood episode, compared with placebo, which appeared largely driven by a significantly lower proportion of patients experiencing a YMRS total score ≥15 or clinical worsening, regardless of whether patients were defined as having earlier-stage BP-I according to age or disease duration at baseline. Based on the changes from baseline in MADRS total scores, treatment with AOM 400 did not appear to worsen depression compared with placebo, using either definition of earlier-stage BP-I. These findings were also shown in the original study, which reported a significant delay in the time to recurrence of any mood episode, and no statistical difference in change from baseline MADRS score between the AOM 400 and placebo groups (Calabrese et al. 2017). The safety profile of AOM 400 was also consistent with the original study, with increased weight and akathisia the most common TEAEs (≥5% of patients) (Calabrese et al. 2017). These findings support the early initiation of AOM 400 maintenance treatment in patients diagnosed with BP-I. By prolonging the time to recurrence of any mood episode, treatment with AOM 400 has the potential to delay functional impairment and other poor outcomes that are associated with disease progression in BP-I (Peters et al. 2016).

There are limitations to this study. Firstly, this analysis is inherently limited by its *post hoc* nature. Secondly, the randomized population for the original study included patients who had been previously stabilized on AOM 400 monotherapy, which may have enriched the population with patients who respond to and tolerate AOM 400 (Calabrese et al. 2017).

We encourage further analysis on the effect of earlier versus later treatment initiation on outcomes in BP-I. Investigating the efficacy of early and sustained intervention with AOM 400 in patients diagnosed with BP-I will deepen our understanding of the potential clinical benefits of early initiation of treatment.

## Conclusion

The findings of this study support the early initiation of AOM 400 maintenance treatment in patients diagnosed with BP-I, which may serve to be clinically beneficial.

## Supplementary Information

Additional file 1.

## Data Availability

To submit inquiries related to Otsuka clinical research, or to request access to individual participant data (IPD) associated with any Otsuka clinical trial, please visit https://clinical-trials.otsuka.com/. For all approved IPD access requests, Otsuka will share anonymized IPD on a remotely accessible data-sharing platform.

## Abbreviations

AE: Adverse event
AOM 400: Aripiprazole once-monthly 400 mg
Ari 2MRTU 960: Aripiprazole 2-month ready-to-use 960 mg
BP-I: Bipolar I disorder
BP-II: Bipolar II disorder
CGI-BP-S: Clinical Global Impression for Bipolar Disorder – Severity
CI: Confidence interval
DSM-IV-TR: Diagnostic and Statistical Manual of Mental Disorders, Fourth Edition, Text Revision
EPS: Extrapyramidal symptoms
HR: Hazard ratio
LAI: Long-acting injectable
MADRS: Montgomery–Åsberg Depression Rating Scale
MMRM: Mixed model for repeated measures
SAE: Serious adverse event
TEAE: Treatment-emergent adverse event
YMRS: Young Mania Rating Scale
US: United States

## References

[CR1] Abilify Asimtufii^®^ (aripiprazole). Prescribing information. Otsuka America Pharmaceutical, Inc., 2023.

[CR2] Abilify Maintena^®^ (aripiprazole). Prescribing information. Otsuka America Pharmaceutical, Inc., 2020.

[CR3] American Psychiatric Association. Diagnostic and Statistical Manual of Mental Disorders, Fourth Edition, Text Revision. Washington, DC: American Psychiatric Association; 2000.

[CR4] American Psychiatric Association. Practice guideline for the treatment of patients with bipolar disorder. 2nd ed. Arlington, VA: American Psychiatric Association; 2010.

[CR5] American Psychiatric Association. Diagnostic and Statistical Manual of Mental Disorders, Fifth Edition, Text Revision. Washington, DC: American Psychiatric Association; 2022.

[CR6] Bartoli F, Bachi B, Calabrese A, et al. Effect of long-acting injectable antipsychotics on emergency department visits and hospital admissions in people with bipolar disorder: a retrospective mirror-image analysis from the Northern Milan Area Cohort (NOMIAC) study. J Affect Disord. 2022;318:88–93. 36058358 10.1016/j.jad.2022.08.096

[CR7] Blanco C, Compton WM, Saha TD, et al. Epidemiology of DSM-5 bipolar I disorder: results from the national epidemiologic survey on alcohol and related conditions–III. J Psychiatr Res. 2017;84:310–7. 27814503 10.1016/j.jpsychires.2016.10.003 PMC7416534

[CR8] Brenner CJ, Shyn SI. Diagnosis and management of bipolar disorder in primary care. Med Clin North Am. 2014;98(5):1025–48. 25134871 10.1016/j.mcna.2014.06.004

[CR9] Calabrese JR, Sanchez R, Jin N, et al. Efficacy and safety of aripiprazole once-monthly in the maintenance treatment of bipolar I disorder: a double-blind, placebo-controlled, 52-week randomized withdrawal study. J Clin Psychiatry. 2017;78(3):324–31. 28146613 10.4088/JCP.16m11201

[CR10] Chakrabarty T, Torres IJ, Bond DJ, Yatham LN. Inflammatory cytokines and cognitive functioning in early-stage bipolar I disorder. J Affect Disord. 2019;245:679–85. 30447566 10.1016/j.jad.2018.11.018

[CR11] Chen AT, Nasrallah HA. Neuroprotective effects of the second generation antipsychotics. Schizophr Res. 2019;208:1–7. 30982644 10.1016/j.schres.2019.04.009

[CR12] Cirone C, Secci I, Favole I, et al. What do we know about the long-term course of early onset bipolar disorder? A review of the current evidence. Brain Sci. 2021;11(3):341. 33800274 10.3390/brainsci11030341 PMC8001096

[CR13] Clemente AS, Diniz BS, Nicolato R, et al. Bipolar disorder prevalence: a systematic review and meta-analysis of the literature. Braz J Psychiatry. 2015;37(2):155–61. 25946396 10.1590/1516-4446-2012-1693

[CR14] Cotton SM, Berk M, Jackson H, et al. Improving functional outcomes in early-stage bipolar disorder: the protocol for the REsearch into COgnitive and behavioural VERsatility trial. Early Interv Psychiatry. 2019;13(6):1470–9. 30740882 10.1111/eip.12797

[CR15] Craddock N, Sklar P. Genetics of bipolar disorder. Lancet. 2013;381(9878):1654–62. 23663951 10.1016/S0140-6736(13)60855-7

[CR16] Grewal M, Dragon J, Golub JS. Age-related disparities in the treatment of borderline/mild hearing loss in the United States. OTO Open. 2022;6(1):2473974X221083092. 35274074 10.1177/2473974X221083092 PMC8902192

[CR17] He L, Xie X, Xue J, et al. Association of the systemic immune-inflammation index with all-cause mortality in patients with arteriosclerotic cardiovascular disease. Front Cardiovasc Med. 2022;9:952953. 36172591 10.3389/fcvm.2022.952953 PMC9510918

[CR18] He P, Ye Z, Liu M, et al. Association of handgrip strength and/or walking pace with incident chronic kidney disease: a UK biobank observational study. J Cachexia Sarcopenia Muscle. 2023;14(2):805–14. 36708151 10.1002/jcsm.13180 PMC10067488

[CR19] Innala L, Berglin E, Möller B, et al. Age at onset determines severity and choice of treatment in early rheumatoid arthritis: a prospective study. Arthritis Res Ther. 2014;16(2):R94. 24731866 10.1186/ar4540 PMC4060263

[CR20] Lähteenvuo M, Paljärvi T, Tanskanen A, et al. Real-world effectiveness of pharmacological treatments for bipolar disorder: register-based national cohort study. Br J Psychiatry. 2023;223(4):456–64. 37395140 10.1192/bjp.2023.75 PMC10866673

[CR21] Narasimhan M, Bruce TO, Masand P. Review of olanzapine in the management of bipolar disorders. Neuropsychiatr Dis Treat. 2007;3(5):579–87. 19300587 PMC2656294

[CR22] Nasrallah HA, Chen AT. Multiple neurotoxic effects of haloperidol resulting in neuronal death. Ann Clin Psychiatry. 2017;29(3):195–202. 28738100

[CR23] Patel M, Jain R, Tohen M, et al. Efficacy of cariprazine in bipolar I depression across patient characteristics: a post hoc analysis of pooled randomized, placebo-controlled studies. Int Clin Psychopharmacol. 2021;36(2):76–83. 33230026 10.1097/YIC.0000000000000344 PMC7846289

[CR24] Peters AT, West AE, Eisner L, et al. The burden of repeated mood episodes in bipolar I disorder: results from the national epidemiological survey on alcohol and related conditions. J Nerv Ment Dis. 2016;204(2):87–94. 26588078 10.1097/NMD.0000000000000425 PMC4733595

[CR25] Poranen J, Koistinaho A, Tanskanen A, et al. Twenty-year medication use trends in first-episode bipolar disorder. Acta Psychiatr Scand. 2022;146(6):583–93. 36177718 10.1111/acps.13504 PMC9828455

[CR26] Quiroz JA, Yatham LN, Palumbo JM, et al. Risperidone long-acting injectable monotherapy in the maintenance treatment of bipolar I disorder. Biol Psychiatry. 2010;68(2):156–62. 20227682 10.1016/j.biopsych.2010.01.015

[CR27] Ratheesh A, Hett D, Ramain J, et al. A systematic review of interventions in the early course of bipolar disorder I or II: a report of the International Society for Bipolar Disorders Taskforce on early intervention. Int J Bipolar Disord. 2023;11(1):1. 36595095 10.1186/s40345-022-00275-3 PMC9810772

[CR28] Risperdal Consta^®^ (risperidone). Prescribing information. Janssen Pharmaceuticals, Inc., 2021.

[CR29] Shi W, Su L, Wang J, et al. Correlation between dietary selenium intake and stroke in the National Health and Nutrition Examination Survey 2003–2018. Ann Med. 2022;54(1):1395–402. 35594240 10.1080/07853890.2022.2058079 PMC9132435

[CR30] Stip E, Javaid S, Bayard-Diotte J, et al. Use of long acting antipsychotics and relationship to newly diagnosed bipolar disorder: a pragmatic longitudinal study based on a Canadian health registry. Ther Adv Psychopharmacol. 2020;10:2045125320957118. 32974000 10.1177/2045125320957118 PMC7493262

[CR31] Tohen M, Goldberg JF, Hassoun Y, Sureddi S. Identifying profiles of patients with bipolar I disorder who would benefit from maintenance therapy with a long-acting injectable antipsychotic. J Clin Psychiatry. 2020;81(4):OT19046AH1. 10.4088/JCP.OT19046AH1 32558403

[CR32] Verdolini N, Hidalgo-Mazzei D, Del Matto L, et al. Long-term treatment of bipolar disorder type I: a systematic and critical review of clinical guidelines with derived practice algorithms. Bipolar Disord. 2021;23(4):324–40. 33354842 10.1111/bdi.13040

[CR33] Vieta E, Montgomery S, Sulaiman AH, et al. A randomized, double-blind, placebo-controlled trial to assess prevention of mood episodes with risperidone long-acting injectable in patients with bipolar I disorder. Eur Neuropsychopharmacol. 2012;22(11):825–35. 22503488 10.1016/j.euroneuro.2012.03.004

[CR34] Vieta E, Salagre E, Grande I, et al. Early intervention in bipolar disorder. Am J Psychiatry. 2018;175(5):411–26. 29361850 10.1176/appi.ajp.2017.17090972

[CR35] Vöhringer PA, Perlis RH. Discriminating between bipolar disorder and major depressive disorder. Psychiatr Clin North Am. 2016;39(1):1–10. 26876315 10.1016/j.psc.2015.10.001

[CR36] Wong SCY, Ng MCM, Chan JKN, et al. Altered risk-taking behavior in early-stage bipolar disorder with a history of psychosis. Front Psychiatry. 2021;12: 763545. 34867547 10.3389/fpsyt.2021.763545 PMC8637446

[CR37] Yan T, Greene M, Chang E, et al. Medication adherence and discontinuation of aripiprazole once-monthly 400 mg (AOM 400) versus oral antipsychotics in patients with schizophrenia or bipolar I disorder: a real-world study using US claims data. Adv Ther. 2018;35(10):1612–25. 30206822 10.1007/s12325-018-0785-y PMC6182631

[CR38] Yatham LN, Kennedy SH, Parikh SV, et al. Canadian Network for Mood and Anxiety Treatments (CANMAT) and International Society for Bipolar Disorders (ISBD) 2018 guidelines for the management of patients with bipolar disorder. Bipolar Disord. 2018;20(2):97–170. 29536616 10.1111/bdi.12609 PMC5947163

