# Supplementary material for: Aripiprazole once-monthly for the treatment of adult patients with earlier-stage bipolar I disorder: a post hoc analysis of data from a double-blind, placebo-controlled, 52-week randomized withdrawal trial
Source: Int J Bipolar Disord. 2024 Oct 27;12:37. doi: 10.1186/s40345-024-00358-3 (PMC11513778; doi:10.1186/s40345-024-00358-3)
Supplement: Supplementary file 1 — Additional file 1. [file 40345_2024_358_MOESM1_ESM.pdf]

Supplementary information

Aripiprazole once-monthly for the treatment of adult patients with earlier-stage bipolar I disorder – a *post hoc* analysis of data from a double-blind, placebo-controlled, 52-week randomized withdrawal trial

Authors

Karimah S. Bell Lynum,[1] Christine F. Castro,[2] Zhen Zhang,[1] Mehul Patel,[1] Mauricio Tohen[3]

Affiliations

1. Otsuka Pharmaceutical Development & Commercialization, Inc., Princeton, NJ, USA

2. Lundbeck LLC, Deerfield, IL, USA

3. Department of Psychiatry & Behavioral Sciences, University of New Mexico, Albuquerque, NM, USA

Corresponding author

Name: Karimah S. Bell Lynum

E-mail address: Karimah.Lynum@otsuka-us.com

Supplementary Table S1: List of institutional review boards (IRBs) according to site number

Trial Site Number	IRB Name	IRB Chair Name
Central IRB - Canada<br>Sites 080, 081	Schulman Associates Institutional Review Board<br>4445 Lake Forest Drive, Suite 300, Cincinnati, Ohio 45242	Sharon Lynn Nelson, MSN, RN, CNS
Local IRB - Japan<br>Site 300	Yuge Hospital Institutional Review Board<br>679-2 Yuge, Tatsuda-machi, Kita-ku, Kumamoto prefecture 861-8002, Japan	Midori Suematsu
Local IRB - Japan<br>Sites 301, 317	Arakaki Hospital Institutional Review Board<br>4-10-3 Ageda, Okinawa, 904-0012, Japan	Norifumi Kunimoto
Local IRB - Japan<br>Site 302	South Toyama Nakagawa Institutional Review Board<br>146 Omachi, Toyama city, Toyama prefecture, 939-8073, Japan	Kazuhiko Katsukawa
Local IRB - Japan<br>Site 303	NHO Tottori Medical Center Institutional Review Board<br>876 Mitsu, NHO Tottori Medical Center, Tottori city, Tottori prefecture, 689-0203, Japan	Tsuruhei Sukegawa
Local IRB - Japan<br>Site 304	Goryokai Medical Corporation Institutional Review Board<br>6-2-3 9-jo, Shinoro, Kita-ku, Sapporo city 002-8029, Hokkaido, Japan	Umeko Sakaoka
Local IRB - Japan<br>Site 305	Asai Clinic Institutional Review Board<br>1-14 Katabira-cho, Hodogaya-ku, Yokohama 240-0013, Kanagawa, Japan	Junichi Kaburaki
Local IRB - Japan<br>Site 306	Asakayama General Hospital Institutional Review Board<br>3-3-16 Imaike-cho, Sakai-ku, Sakai city 590-0018, Osaka, Japan	Ryo Takahashi
Local IRB - Japan<br>Site 308	Nara Medical University Institutional Review Board<br>840 Shijo-cho, Kashihara 634-8522, Nara, Japan	Masatoshi Hasegawa

Trial Site Number	IEC/IRB Name	IEC/IRB Chair Name
Local IRB - Japan<br>Site 309	Hoshi General Hospital Institutional Review Board<br>159-1 Mukaikawara-machi, Koriyama 963-8501, Fukushima, Japan	Kaori Hirai
Local IRB - Japan<br>Site 311	Shinagawa East One Medical Clinic Institutional Review Board<br>2-16-1, Konan Minato-ku, Tokyo 108-0075, Japan	Hideaki Sakai
Local IRB - Japan<br>Sites 312, 313	Seiwakai Medical Corporation Association Yutaka Clinic Institutional Review Board<br>3-14-20 Sagamiono, Minami-ku, Sagamihara-shi 252-0303, Kanagawa, Japan	Soichiro Watanabe
Local IRB - Japan<br>Site 314	Tokyo Women's Medical University Institutional Review Board<br>8-1 Kawada-cho, Shinjyuku-ku 162-8666, Tokyo, Japan	Jun Ishigooka
Local IRB - Japan<br>Site 315	Medical Corporation Kyouwakai, Hannan Hospital Institutional Review Board<br>277, Handa Minamino-cho, Naka-ku, Sakai 599-8263, Osaka, Japan	Tetsuya Inoue
Local IRB - Japan<br>Site 318	Medical Corporation Houmankai Umezu Clinic Institutional Review Board<br>2-6-12 Harada, Chikushino 818-0024, Fukuoka, Japan	Tetsuji Inou
Local IRB - Japan<br>Site 319	Takeda General Hospital Institutional Review Board<br>3-27 Yamagamachi, Aizu Wakamatsu 965-8585, Fukushima, Japan	Kouichi Osonoe
Local IRB - Japan<br>Site 320	Fukuoka University Hospital Institutional Review Board<br>7-45-1 Nanakuma, Jyonan-ku, Fukuoka-shi 814-0180, Fukuoka, Japan	Toshihiko Yanase
Local IRB - Japan<br>Site 321	Iwate Medical University Institutional Review Board<br>19-1m Uchimaru, Morioka-shi 020-8505, Iwate, Japan	Toru Sugiyama

Trial Site Number	IEC/IRB Name	IEC/IRB Chair Name
Local IRB - Japan<br>Site 322	National Hospital Organization Hizen Psychiatric Center Institutional Review Board<br>160, Mitsu, Yoshinogari-cho, Kanzaki-gun 842-0192, Saga, Japan	Kijiro Hashimoto
Local IRB - Japan<br>Site 323	Nagasaki Medical Center of Psychiatry Institutional Review Board<br>1575-2 Seibu Machi, Omura 856-0847, Nagasaki, Japan	Kyoko Kubo
Central IRB - Poland<br>Sites 100, 102, 103, 104	Komisja Bioetyczna przy Bydgoskiej lzbie Lekarskiej<br>ul. Powstadcow Warszawy 1 1<br>85-681 Bydgoszcz	Chair Name not provided
Central IRB - Romania<br>Sites 150, 151, 152, 153, 154, 155	Comisia Nationala de Etica pentru Studiul Clinic al Medicamentului<br>Str. Av. Sanatescu nr.48<br>Sector I<br>Bucuresti	Chair Name not provided
Local IRB - South Korea<br>Site 250	Institutional Review Board of The Catholic University of Korea<br>10, 63-ro, Yeongdeungpo-gu, 150-713, Republic of Korea	Ki-Sung Ryu
Local IRB - South Korea<br>Site 251	Institutional Review Board of Eulji General Hospital<br>68, Hangeulbiseok-ro, Nowon-gu, Seoul, 139-711 Republic of Korea	Kwon, O Hyun
Local IRB - South Korea<br>Site 252	Institutional Review Board of Jeju National University Hospital<br>Aran 13gil 15, Jeju-si, Jeju Special Self-Governing Province, 690-767, Republic of Korea	Lee, Chang Sub
Local IRB - South Korea<br>Site 253	Institutional Review Board of Chungnam National University Hospital<br>282, Munhwa-ro, Jung-gu, Daejeon, 30”1-721, Republic of Korea	Suhm Kwang-Sun

Trial Site Number	IEC/IRB Name	IEC/IRB Chair Name
Local IRB - South Korea<br>Site 254	Institutional Review Board of Korea University Anam Hospital<br>73 Inchon-ro, Seongbuk-Gu, Seoul, 136-705, Republic of Korea	Jeen, Yoon Tae
Local IRB - South Korea<br>Site 255	Institutional Review Board of Dongguk University Ilsan Hospital<br>27 Dongguk-rom, Ilsandong-gu, Goyang-si, 410-773, Republic of Korea<br>Site 255	Kim Eung-Jung
Local IRB - South Korea<br>Site 256	Institutional Review Board of Hallym University Sacred Heart Hospital<br>22, Gwanpyeong-ro 170beon-gil, Dongan-gu, Anyang-si, Gyeonggi-do, 431-070, Republic of Korea	Kim Kwang Nam
Local IRB - Taiwan<br>Site 200	Taipei City Hospital Institutional Review Board<br>No. 145, Zhengzhou Road, Taipei City, 103 Taiwan	Kuang-Shen Li
Local IRB - Taiwan<br>Sites 201, 202	Chang Gung Medical Foundation Institutional Review Board<br>No. 199, Tunhua North Road, Taipei City, 105 Taiwan	Tsang Tang
Central IRB - United States<br>Sites 001, 002, 003, 004, 005, 006, 007, 008, 009, 010, 011, 012, 013, 015, 016, 017, 019, 020, 021, 022, 023, 025, 027, 028, 029, 030, 033, 034, 035, 036, 037, 038, 039, 042, 043, 045, 046, 048, 049, 052, 053, 054, 055, 056, 057, 058, 059, 060, 061, 064, 065, 066, 067, 068, 069	Schulman Associates Institutional Review Board<br>4445 Lake Forest Drive, Suite 300, Cincinnati, Ohio 45242	Sharon Lynn Nelson, MSN, RN, CNS
Local IRB - United States<br>Site 014	UC Irvine: Office of Research Institutional Review Board<br>5171 California, Suite 150, Irvine, California 92697	Kenneth G. Linden, M.D., Ph.D.
Local IRB - United States<br>Site 024	University at Buffalo: Health Sciences Institutional Review Board<br>875 Ellicott Street, Buffalo, New York 14203	Ron Moscati

**Trial Site Number**	**IEC/IRB Name**	**IEC/IRB Chair Name**
Local IRB - United States<br>Sites 040, 051	Western Institutional Review Board<br>3535 7th Avenue SW, Olympia, Washington 98502	Bert Wilkins, J.D., MHA
Local IRB - United States<br>Site 047	Louisiana State University Health Sciences Institutional Review Board<br>433 Bolivar Street Suite 206, New Orleans, Louisiana 70112	Kenneth E. Kratz, Ph.D.
